# CRISPR-mediated multiplexed genetic manipulation

**DOI:** 10.18632/oncotarget.13371

**Published:** 2016-11-15

**Authors:** Lin Lin, Johan Vad-Nielsen, Yonglun Luo

**Affiliations:** Department of Biomedicine, Aarhus University, Aarhus C, Denmark

**Keywords:** CRISPR, Cas9, genome editing, Golden Gate Assembly, orthogonal genetic manipulation

The relative ease of using Clustered Regularly Interspaced Short Palindromic Repeats (CRISPR)- associated proteins (Cas-proteins) in genome editing has rapidly made this RNA-guided endonuclease system the method of choice in almost all current applications involving genetic manipulations, with Cas9 from *S. pyogenes* (SpCas9) being the most well characterized and broadly used [1]. Compared to the early generations of programmable DNA endonucleases such as the Zinc-finger nuclease (ZFN) or Transcription Activator-Like Effector Nuclease (TALEN), the CRISPR-Cas9 system is time-, labor-, and cost-effective. Mediated by a small guide RNA (sgRNA or gRNA), Cas9 can specifically introduce double-strand DNA breaks at pre-selected genomic loci which (i) contain a target sequence (also known as a protospacer) complementary to the typically 20-nt guide sequence of the gRNA, and (ii) are followed by a protospacer sequence adjacent motif (PAM, 5’-NGG- 3’ for SpCas9). Furthermore, by co-delivery of Cas9 and multiple gRNAs, the CRISPR/Cas9 system enables the simultaneous modification of several loci/genes in the same cell [2]. Currently, most CRISPR-Cas9-based studies rely on plasmid-based or viral vector mediated expression of the Cas9 and gRNAs. The generation of plasmids expressing multiple gRNAs has been a time-consuming procedure involving multiple steps of cloning. To facilitate CRISPR-mediated multiplexed gene editing applications, we have opted for the Golden-Gate assembly method and developed a system for rapid construction of plasmids expressing up to 30 gRNAs within one week [3].

Golden-Gate assembly has been an attractive and cost-effective method for rapidly assembly of multiple DNA fragments in a single reaction. This method uses a special class of enzymes (type IIS restriction enzymes) which recognize asymmetric DNA sequences and induce staggered DNA-cleavage outside of their recognition sites. Three type IIS restriction enzymes have been favored for Golden-Gate assembly: *BbsI* (5’-GAAGAC(2/6); i.e. it cleaves at 2 and 6 nt downstream of the sense strand and antisense strand, respectively, and creates a 4-nt 5’ overhang), *BsaI* (5’-GGTCTC(1/5)), and *BsmbI* (5’-CGTCTC(1/5)) [4]. These three enzymes have 6 nt recognition sites, thus minimizing the frequency of such sites in genomic DNA (approximately one recognition site in 4^6^ nt). Furthermore, the generation of 4-nt overhangs can in principle allow for the assembly of up to 128 (4X4X4X2) different DNA fragments in one step. To minimize the number of plasmids, we utilized a three-step Golden-Gate assembly (GGA) strategy to generate a vector carrying up to 30 gRNA expression cassettes: First, double-strand gRNA guide oligonucleotides with 4-nt 5’ overhangs (5’-CACC sense strand, 5’-AAAC antisense strand) are cloned to a set of single gRNA expression modular plasmids using *BbsI*-mediated GGA; Secondly, up to ten modular gRNA expression plasmids are assembled into a second modular plasmid using *BsaI*-mediated GGA to generate a plasmid expressing 2 to 10 gRNAs; finally up to three such secondary modular plasmids can be assembled to generate a final plasmid using *BsmBI*-mediated GGA and thus generating a single plasmid expressing up to 30 gRNAs.

A proof-of-concept validation of the multiplexed gRNA expression array has been conducted in CRISPR-based simultaneous gene knockout and inhibitions using our dual-fluorescent reporter system (C-Check) and in human cells [3, 5]. However, the system is in principle compatible with all applications that employ simultaneous delivery of multiple SpCas9 gRNAs into one cell, such as CRISPR-mediated epigenetic modification [6] or gene activation (CRISPRa) [7]. Studies by Konermann S., *et al.* [7] and us (Xiong K. *et al.* submitted) show that the synergistic activation mediator composed of a combination of gRNA 2.0, NLS-dCas9-VP64 and MS2- p65-HSF1 is the most effective transcriptional activation system developed. To further expand the applicability of our multiplexed gRNA expression array system, we have generated another set of modular plasmids that encode a single gRNA 2.0, which contains MS2 loops at the tetraloop and stemloop 2 of the gRNA scaffold (unpublished results).

**Figure 1:**
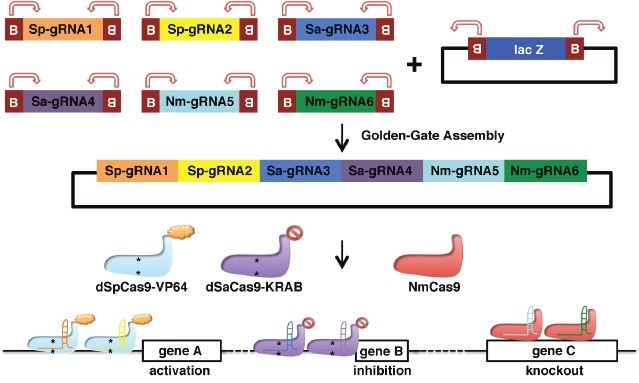
Schematic illustration of simultaneous and orthogonal gene inhibition, activation, and knockout using Cas9 orthologues and multiplexed gRNA expression array Six modular gRNA expression plasmids, with two gRNAs for each Cas9 orthologs (SpCas9, SaCas9, and NmCas9), are assembled into one multiplexed gRNA expression array based on *Bsal*-mediated Golden- Gate Assembly. Letter “B” and arrows indicated the *Bsal* recognition site and cleavage position, respectively. Co-delivery of the multiplexed gRNA expression array with the nuclease-deficient SpCas9 fused to transcriptional activation domain VP64 (dSpCas9-VP64), nuclease-deficient SaCas9 fused to transcriptional repression domain KRAB (dSaCas9-KRAB), and wild type NmCas9 enables simultaneous and orthogonal activation of gene **A**., inhibition of gene **B**., and knockout of gene **C.** Asterisks (*) represent nuclease-deficient mutations.

Other applications that may benefit from our multiplexed gRNA expression array are simultaneous orthogonal genetic manipulations, such as gene inhibition, activation, and knockout. Several other Cas9 orthologues have been identified such as Cas9 from *Neisseria meningitides* (NmCas9), *Staphylococcus aureus* (SaCas9), and Cpf1 [8]. These Cas9 orthologues have efficient genome-editing activity but bind a variety of PAM and use different gRNA scaffolds. To further expand the multiplexed gRNA expression array kit, future studies should be conducted to generate gRNA expression modular plasmids for each Cas9 ortholog. Co-delivery of different Cas9 orthologues along with the corresponding gRNAs will allow simultaneous, orthogonal, and sophisticated genetic manipulations and facilitate the study of genetic circuits (Figure 1).

To facilitate the broad application of our method, all plasmids developed in this study as well as those developed in the future have been made publically available through the nonprofit global plasmid repository Addgene (https://www.addgene.org/Yonglun_Luo/). A more detailed step-by-step protocol will be presented through Bio-protocol. In addition to the above-mentioned improvements, multiplexed gRNA expression array plasmids driven by different promoters will also be generated in future studies.
